# Just the facts: diagnosis and management of Stevens–Johnson syndrome/toxic epidermal necrolysis

**DOI:** 10.1007/s43678-025-01041-x

**Published:** 2026-01-06

**Authors:** Jensen Yeung, Marlene Dytoc, Eric Mutter, Jan P. Dutz

**Affiliations:** 1 Department of Dermatology, Women’s College Hospital, Toronto, ON Canada; 2 Department of Dermatology, Sunnybrook Health Sciences Centre, Toronto, ON Canada; 3 Division of Dermatology, Department of Medicine, University of Toronto, Toronto, ON Canada; 4 Probity Medical Research, Waterloo, ON Canada; 5 Division of Dermatology, Department of Medicine, University of Alberta, Edmonton, AB Canada; 6 Department of Emergency Medicine, The Ottawa Hospital, Ottawa, ON Canada; 7 Department of Emergency Medicine, University of Ottawa, Ottawa, ON Canada; 8 Department of Dermatology and Skin Science, BC Children’s Hospital Research Institute, University of British Columbia, Vancouver, BC Canada

**Keywords:** Stevens–Johnson syndrome, Toxic epidermal necrolysis, Mucocutaneous, Emergency department, Syndrome de Stevens-Johnson, Nécrolyse épidermique toxique, Mucocutanée, Service des urgences

## Clinical scenario

A 70-year-old male presented to the ED with a 3-day history of fever, sore throat, and myalgia. Vital signs were notable for a temperature of 38.6 °C, heart rate of 110 beats per minute, blood pressure of 100/60 mmHg, and oxygen saturation of 95% on room air. The patient’s laboratory values were largely within normal limits: white blood cell count 10.1 × 10⁹/L, creatinine 50 μmol/L, urea 3.8 mmol/L, fasting glucose 5.0 mmol/L, aspartate aminotransferase 35 U/L, and alanine aminotransferase 29 U/L. C-reactive protein was elevated at 30 mg/L. Immunoglobulin levels were within reference ranges. Physical examination revealed erythematous plaques, bullae, and epidermal sloughing involving 8% of total body surface area including the trunk and limbs. Several erosions were also noted inside the buccal mucosa. The patient was diagnosed with hyperuricemia 4 weeks ago and was prescribed oral allopurinol 300 mg qd.

## Key clinical questions

### What are the main pathogenic hallmarks of Stevens–Johnson syndrome/toxic epidermal necrolysis?

Stevens–Johnson syndrome and toxic epidermal necrolysis are rare and life-threatening mucocutaneous immune reactions, predominantly drug-induced, and characterized by severe epidermal necrosis and sloughing [1]. Stevens–Johnson syndrome/toxic epidermal necrolysis is a disease continuum with 3 categories that are distinguished by total body surface area of epidermal detachment: Stevens–Johnson syndrome (< 10% total body surface area), Stevens–Johnson syndrome/toxic epidermal necrolysis overlap (10% < total body surface area < 30%), and toxic epidermal necrolysis (> 30% total body surface area) [1, 2]. Stevens–Johnson syndrome/toxic epidermal necrolysis pathogenesis involves direct drug binding to major histocompatibility complex class I molecules and T cell receptors [2]. Subsequent activation of drug-specific cytotoxic CD8 + T lymphocytes, producing cytolytic Fas ligand and granulysin, leads to keratinocyte and mucosal cell necrosis [1, 2].

### What are the clinical features of Stevens–Johnson syndrome/toxic epidermal necrolysis?

Stevens–Johnson syndrome/toxic epidermal necrolysis symptoms develop 3–4 weeks after exposure to the causative drug, although shorter (4 days) and longer (8 weeks) durations have been reported [3]. The acute phase of Stevens–Johnson syndrome/toxic epidermal necrolysis may begin with a prodrome of fever and influenza-like symptoms (cough, sore throat, malaise, myalgia) that precede mucocutaneous manifestations by 1–3 days [1, 2]. Skin pain and skin peeling with involvement of the mucosae are the cardinal symptoms. Lesions initially develop as erythematous or violaceous coalescing macules with purpuric centers that may spread to involve other areas [2]. Atypical targetoid lesions with dark centers may also be present [2, 3]. Vesicles and bullae eventually form, leading to extensive and painful epidermal sloughing resembling thermal injury [1, 2]. Erosion of mucosal membranes, as mentioned—including the oral and nasal cavities, conjunctivae, and genitourinary tract—occurs in approximately 90% of Stevens–Johnson syndrome/toxic epidermal necrolysis cases [2, 3]. Importantly, airway involvement may result from mucosal erosions or bronchial epithelial sloughing and can rapidly lead to respiratory compromise. Early airway assessment in the ED is therefore essential.

### What are the main triggers of Stevens–Johnson syndrome/toxic epidermal necrolysis?

The urate-lowering drug allopurinol is a common trigger of Stevens–Johnson syndrome/toxic epidermal necrolysis [4]. The Human Leukocyte Antigen-B*58:01 allele is strongly correlated with allopurinol-induced severe cutaneous adverse reactions [5]. The American College of Rheumatology conditionally recommends testing Human Leukocyte Antigen-B*58:01 before starting allopurinol for patients of Southeast Asian descent (Han Chinese, Korean, Thai) and African American patients, who have a higher prevalence of Human Leukocyte Antigen-B*58:01 [6]. While testing recommendations are based on cost-effectiveness across populations, Human Leukocyte Antigen-B*58:01 remains a risk factor for allopurinol hypersensitivity reactions regardless of ethnicity. Other Stevens–Johnson syndrome/toxic epidermal necrolysis causative drugs include anticonvulsants, antibiotics, sulfonamides, antiretrovirals, and oxicam nonsteroidal anti-inflammatory drugs [2, 4]. *Mycoplasma pneumoniae* infection was historically considered a common trigger of Stevens–Johnson syndrome/toxic epidermal necrolysis, especially in children [2]. However, recent evidence suggests that *Mycoplasma pneumoniae*–induced rash and mucositis (now often classified as a reactive infectious mucocutaneous eruption) has a distinct pathophysiology and typically follows a milder clinical course compared to more severe drug-induced Stevens–Johnson syndrome/toxic epidermal necrolysis [7].

### How is Stevens–Johnson syndrome/toxic epidermal necrolysis diagnosed?

Urgent diagnosis and treatment is necessary as Stevens–Johnson syndrome/toxic epidermal necrolysis can lead to life-threatening complications including acute respiratory failure, hypovolemic shock, sepsis, and septic shock [2]. A dermatology consultation should be immediately sought to confirm diagnosis and initiate appropriate treatment. However, many community hospitals across Canada currently lack dermatology consultation services or face significant delays. In such cases, acute management may be led by internists, pediatricians, in consultation with clinical pharmacologists. Regardless of specialty, clinicians with experience in evaluating and managing Stevens–Johnson syndrome/toxic epidermal necrolysis should oversee care, particularly given the increasing use of targeted immunomodulatory therapies.

Diagnosis relies on clinical and histological features [1]. A complete medication history is required as early cessation of the offending agent significantly improves prognosis [2]. A complete physical examination of the skin, eyes, oral and nasal cavities, and genitourinary system is needed. Laboratory tests are needed to assess for infection, and hepatic and renal function, and include a complete blood count with differential, metabolic panel, erythrocyte sedimentation rate, and C-reactive protein [2, 3]. Bacterial/fungal cultures from blood and target lesions should be considered given the high risk of infection [2]. A chest radiograph may be warranted given the high risk of pneumonia [2]. *Mycoplasma pneumoniae* screening tests (serology and/or polymerase chain reaction) should be considered in children and young adults under 21 years of age, as *Mycoplasma pneumoniae*–associated mucocutaneous eruptions are uncommon in older individuals [2]. Skin biopsy is not routinely performed in EDs, but histopathological analysis revealing apoptotic keratinocytes and infiltrating T lymphocytes may help confirm a Stevens–Johnson syndrome/toxic epidermal necrolysis diagnosis [2, 3].

### What are the differential diagnoses of Stevens–Johnson syndrome/toxic epidermal necrolysis?

Differential diagnoses of Stevens–Johnson syndrome/toxic epidermal necrolysis include erythema multiforme, acute generalized exanthematous pustulosis, generalized bullous fixed drug eruption, phototoxic eruptions, staphylococcal scalded skin syndrome, paraneoplastic pemphigus, and linear Immunoglobulin A bullous dermatosis [2]. We note that while mucositis is a hallmark of Stevens–Johnson syndrome/toxic epidermal necrolysis, it is typically extensive, inflammatory, and often hemorrhagic. Mild lip swelling or isolated targetoid lesions are more consistent with erythema multiforme and should not be mistaken for Stevens–Johnson syndrome/toxic epidermal necrolysis.

### What are the management steps of Stevens–Johnson syndrome/toxic epidermal necrolysis patients in the ED?

**Fig. 1:**
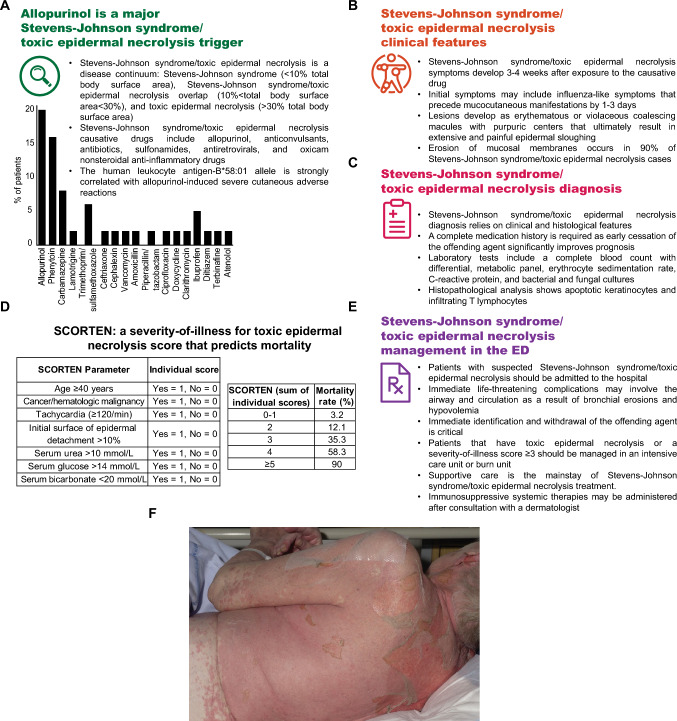
**A** Allopurinol is the most common Stevens–Johnson syndrome/toxic epidermal necrolysis drug trigger [4]. **B** Typical clinical progression of Stevens–Johnson syndrome/toxic epidermal necrolysis from a prodrome of fever and influenza-like symptoms to mucocutaneous involvement. **C** Initial diagnostic workup and management priorities in suspected Stevens–Johnson syndrome/toxic epidermal necrolysis cases. **D** Severity-of-illness for toxic epidermal necrolysis: a severity-of-illness score that predicts mortality in Stevens–Johnson syndrome/toxic epidermal necrolysis patients [9]. **E** Emergency care priorities and inpatient management of Stevens–Johnson syndrome/toxic epidermal necrolysis. F. Severe epidermal sloughing in a patient with toxic epidermal necrolysis

No consensus guidelines exist for the medical management of Stevens–Johnson syndrome/toxic epidermal necrolysis, although there are recommendations for supportive care [8]. Patients with suspected Stevens–Johnson syndrome/toxic epidermal necrolysis should be admitted to the hospital [3]. Emergency physicians must identify any immediate life-threatening complications that may involve the airway and circulation as a result of bronchial erosions and hypovolemia [3]. Sepsis is the leading cause of death in Stevens–Johnson syndrome/toxic epidermal necrolysis patients and must be considered [3]. Initial emergency care may include administration of balanced crystalloids to restore euvolemia and stabilize the patient [3]. Immediate identification and withdrawal of the offending agent is a critical next step. Dermatologists should be directly involved in patient management, together with experts in treating complications of severe epidermal loss [8]. Disease severity and prognosis can be assessed using the severity-of-illness for toxic epidermal necrolysis score (also known as SCORTEN) (Fig. 1) [9]. The severity-of-illness for toxic epidermal necrolysis score—based on 7 readily available clinical parameters—helps predict mortality and guide the level of care. Patients with mild disease (score 0–1) and limited skin involvement may be managed on non-specialized wards. Patients with higher severity-of-illness for toxic epidermal necrolysis scores (≥ 3) or > 30% total body surface area involvement should be admitted to an intensive care unit or burn unit where multidisciplinary expertise is available to monitor for and manage complications such as sepsis and respiratory failure [1]. Supportive care includes wound care, ocular care, oral care, urogenital care, pain management, and stress ulcer prophylaxis [8]. Adjunctive systemic therapies, including cyclosporine, tumor necrosis factor-alpha blockers, Janus kinase inhibitors, intravenous immunoglobulin, and plasmapheresis, have shown efficacy in small trials but remain controversial, and use should be deferred to the admitting physician [3, 10].

## Case resolution

Complete medication history did not reveal any newly ingested medications apart from allopurinol. The patient was admitted, and allopurinol was immediately discontinued upon suspicion of Stevens–Johnson syndrome/toxic epidermal necrolysis. Blood and bacterial cultures were negative. Upon consultation with a dermatologist, the patient was diagnosed with Stevens–Johnson syndrome (< 10% total body surface area). The patient was started on cyclosporine (5 mg/kg/d for 10 days), and optimal wound care was provided. Complete resolution of symptoms was achieved after 6 weeks.

## Key points

1. Stevens–Johnson syndrome and toxic epidermal necrolysis are rare mucocutaneous immune reactions, predominantly drug-induced, and characterized by severe epidermal sloughing.
2. Stevens–Johnson syndrome/toxic epidermal necrolysis symptoms could develop 4 days–8 weeks after exposure to the causative drug. Influenza-like symptoms may precede mucocutaneous manifestations.
3. Diagnosis relies on clinical and histological features. Lesions develop as erythematous or violaceous coalescing patches or plaques that could become purpuric leading to bullae that eventually desquamate. Erosion of mucosal membranes occurs in 90% of Stevens–Johnson syndrome/toxic epidermal necrolysis cases.
4. Stevens–Johnson syndrome/toxic epidermal necrolysis is a life-threatening medical emergency that can deteriorate rapidly and requires urgent, multidisciplinary care. Management includes close monitoring, supportive care, and consideration of systemic immunosuppressive therapies, with readiness to escalate to intensive care unit-level or burn unit care if needed.

## Declarations

### Patient photographs

The patient photograph shown in Fig. 1F was kindly provided by Dr. Robert Gniadecki with patient’s consent.
